# Inhibition of human macrophage activation *via* pregnane neurosteroid interactions with toll-like receptors: Sex differences and structural requirements

**DOI:** 10.3389/fimmu.2022.940095

**Published:** 2022-07-29

**Authors:** Irina Balan, Laure Aurelian, Kimberly S. Williams, Brian Campbell, Rick B. Meeker, A. Leslie Morrow

**Affiliations:** ^1^ Department of Psychiatry, Department of Pharmacology, Bowles Center for Alcohol Studies, University of North Carolina at Chapel Hill, School of Medicine, Chapel Hill, NC, United States; ^2^ Stanford University School of Medicine, Stanford, CA, United States; ^3^ Department of Neurology, University of North Carolina at Chapel Hill, School of Medicine, Chapel Hill, NC, United States; ^4^ Translational Sciences, Sage Therapeutics Inc., Cambridge, MA, United States

**Keywords:** allopregnanolone (3α, 5α-THP), SGE-516, cytokines, chemokine, neurosteroid (3α, 5α)-3 21-dihydroxypregnan-20-one (3α, 5α-THDOC)

## Abstract

We recently discovered that (3α,5α)3-hydroxypregnan-20-one (allopregnanolone) inhibits pro-inflammatory toll-like receptor (TLR) activation and cytokine/chemokine production in mouse macrophage RAW264.7 cells. The present studies evaluate neurosteroid actions upon TLR activation in human macrophages from male and female healthy donors. Buffy coat leukocytes were obtained from donors at the New York Blood Center (http://nybloodcenter.org/), and peripheral blood mononuclear cells were isolated and cultured to achieve macrophage differentiation. TLR4 and TLR7 were activated by lipopolysaccharide (LPS) or imiquimod in the presence/absence of allopregnanolone or related neurosteroids and pro-inflammatory markers were detected by ELISA or western blotting. Cultured human monocyte-derived-macrophages exhibited typical morphology, a mixed immune profile of both inflammatory and anti-inflammatory markers, with no sex difference at baseline. Allopregnanolone inhibited TLR4 activation in male and female donors, preventing LPS-induced elevations of TNF-α, MCP-1, pCREB and pSTAT1. In contrast, 3α,5α-THDOC and SGE-516 inhibited the TLR4 pathway activation in female, but not male donors. Allopregnanolone completely inhibited TLR7 activation by imiquimod, blocking IL-1-β, IL-6, pSTAT1 and pIRF7 elevations in females only. 3α,5α-THDOC and SGE-516 partially inhibited TLR7 activation, only in female donors. The results indicate that allopregnanolone inhibits TLR4 and TLR7 activation in cultured human macrophages resulting in diminished cytokine/chemokine production. Allopregnanolone inhibition of TLR4 activation was found in males and females, but inhibition of TLR7 signals exhibited specificity for female donors. 3α,5α-THDOC and SGE-516 inhibited TLR4 and TLR7 pathways only in females. These studies demonstrate anti-inflammatory effects of allopregnanolone in human macrophages for the first time and suggest that inhibition of pro-inflammatory cytokines/chemokines may contribute to its therapeutic actions.

## 1 Introduction

Inflammation is associated with the pathogenesis of numerous systemic, neurodegenerative and psychiatric diseases (1–3). Furthermore, inflammation may arise from bacterial and fungal infections (4–7), leading to long-lasting complications of infectious disease, such as long COVID-19 or sepsis (8, 9). The contribution of mononuclear phagocytes (macrophages/microglia) to inflammatory and neuroinflammatory diseases is well documented (10–13), but so too are many pro-survival and repair actions that favor survival and recovery. While strong immune suppressants have been available for decades (14), progress in the development of agents that provide control of specific macrophage functions (specifically inflammation) have lagged behind. The ability to regulate macrophage functions with greater precision is necessary to facilitate efforts to suppress deleterious inflammation while retaining supportive functions.

Toll-like receptors (TLRs) play an important role in various activities of macrophages (15). TLRs belong to a family of pattern recognition receptors that can recognize and respond to molecular signatures referred to as pathogen-associated molecular patterns (PAMPs) and danger-associated molecular patterns (DAMPs) (16). At least 10 human TLRs have been identified (17). TLRs share common structural domains, which define their ability to recruit the adaptor proteins that regulate signaling. Recognition of PAMPs and DAMPs by TLRs initiate signaling pathways that involve phosphorylation (activation) of transcription factors, their translocation to the nucleus and culminate in the production of inflammatory cytokines such as tumor necrosis factor alpha (TNF-α), interleukins 1 and 6 (IL-1; IL-6) and chemokines such as monocyte chemoattractant protein-1 (MCP-1) (5, 15, 18–21). Excessive TLR activation contributes to the development of many inflammatory and neuroinflammatory diseases, such as systemic lupus erythematosus, infection-associated sepsis, atherosclerosis, asthma (22), ischemia (23), depression (24, 25), alcohol use disorders (26, 27), traumatic brain injury (28), neurodegeneration (29, 30), and epilepsy (31, 32).

There is a growing appreciation for potential interactions between systemic immune activation and brain TLRs that facilitate detrimental inflammatory activity. Thus, blocking TLR signals may be useful to regulate overactive systemic and CNS responses (22). We recently discovered that endogenous neurosteroid (3α,5α)3-hydroxypregnan-20-one (allopregnanolone, 3α,5α-THP) inhibits myeloid differentiation primary response 88 (MyD88)-dependent TLR2, TLR4, and TLR7 (but not TIR-domain-containing adapter-inducing interferon-β dependent TLR3) pro-inflammatory signal activation and the production of cytokines/chemokines through its ability to block TLR-MyD88 binding in the mouse macrophage RAW264.7 cell line and the alcohol-preferring (P) rat brain (33, 34). Since inflammatory conditions are ubiquitous hallmarks of human disease (vide infra), it is essential to establish the validity of this work in human macrophages and to examine sex as a biological variable in the studies. Here, we examine the effect of allopregnanolone as well as the endogenous neurosteroid (3α,5α)-3,21-dihydroxypregnan-20-one (3α,5α-THDOC) and a synthetic analog of allopregnanolone, SGE-516, on cultured human monocyte-derived macrophages (hMDM), obtained from both male and female healthy donors.

Allopregnanolone and 3α,5α-THDOC display minimal activity at nuclear genomic receptors (35), but are potent positive modulators of γ-aminobutyric acid type A (GABA_A_) receptors (36–38). They have anesthetic, anticonvulsant, sedative, and anxiolytic effects (39), and modulate the hypothalamic pituitary adrenal axis to reduce stress activation (40). Significantly, allopregnanolone and/or its precursors progesterone and pregnenolone, were shown to be effective in clinical studies of schizophrenia (41) and cocaine craving (42). A proprietary formulation of allopregnanolone, Brexanolone i.v., is a fast-and long-acting antidepressant and the only FDA specifically approved treatment for post-partum depression (43–45). Further, allopregnanolone has putative therapeutic activity in animal models of alcoholism (46–48), traumatic brain injury (49, 50), multiple sclerosis (51, 52), and Alzheimer’s disease (53). The synthetic compound SGE-516, a 1,2,5-triazole analog of allopregnanolone, was reported to display better aqueous solubility while maintaining efficacy as an allosteric modulator at GABA_A_ receptors (54). SGE-516 has been reported to exhibit anticonvulsant activity as demonstrated in experimental seizure and epilepsy animal models (55–57). In addition, SGE-516 has recently been shown to protect mice from chronic stress-induced behavioral deficits including restoration of theta power ratios indicative of normalized network activity in brain (58).

Many effects of neurosteroids have been attributed to actions at GABA_A_ receptors. Here, however, we demonstrate unique inhibitory effects of allopregnanolone on the modulation of pro-inflammatory MyD88-dependent TLR4 and TLR7 signaling pathways in hMDMs resulting in diminished cytokine production. Inhibition of TLR4 pathways was observed in males and females, however inhibition of TLR7 pathways was only observed in hMDM from female subjects. We further observed the structural specificity of allopregnanolone in the inhibition of these signals, suggesting that D ring modifications may be detrimental to the anti-inflammatory efficacy of this class of compounds in both males and females.

## 2 Materials and methods

### 2.1 Culture of hMDMs

Human buffy coat leukocytes were obtained from healthy donors at the New York Blood Center (http://nybloodcenter.org/), a non-profit organization for the collection and distribution of blood for clinical and research purposes. No personal identifiers were sent with the shipment. The NY Blood Center maintains IRB approval for their blood collection procedures and UNC School of Medicine issued an IRB waiver for this work since no personal identifiers were made available to investigators.

Culture of hMDM was as previously described (59–61) with minor modifications. Blood was diluted 2:1 with phosphate buffered saline (PBS), layered on top of Ficoll-Paque (GE Healthcare 17-1440-03), and centrifuged at 500 X g for 20 min. The peripheral blood mononuclear cells (PBMCs) were collected from the PBS/Ficoll-Paque interface and transferred to a tube containing PBS to a total volume of 40 ml. The resulting PBMCs were incubated in red blood cell lysis buffer (Sigma R7757, CSH protocols) to remove any red blood cell contamination. PBS was added to a volume of 40 ml, the PBMCs were re-suspended and centrifuged at 150 X g for 20 min. The wash step was repeated once and the final pellet was re-suspended in Dulbecco’s modified Eagle medium (DMEM) with high glucose, 10% fetal bovine serum (FBS, Gibco 160000-044) and 20 µg/ml gentamicin (Gibco 15750-60). Cells were counted and aliquoted into low adhesion 6 well plates (Corning 3471) at a density of approximately 10^6^ cells/cm^2^. Media was changed 2-3 times weekly to maintain optimal cell heath. Cells were cultured for 5-7 days to allow monocyte attachment, then remaining white blood cells were then washed from the plate, yielding a pure macrophage culture. To minimize any differentiation bias, the adherent cells were grown in complete DMEM without colony stimulating factor (CSF) supplements for one to two weeks to achieve 70-80% coverage of the plate, indicative of macrophage differentiation. Previous studies indicated that the macrophages can secrete significant amounts of both M-CSF and/or GM-CSF to support their own growth (59) although basal GM-CSF is typically very low.

### 2.2 Immune protein profile of macrophage conditioned medium

Basal characteristics of hMDM cultured under the above conditions were determined in the initial establishment of the culture protocol. Medium was collected and centrifuged at 1000 X g for 5 min to remove any floating cells or debris in the medium. The cell free medium was added to a RayBiotech human antibody array L-507 (RayBiotech Life, Inc., Peachtree, GA) and processed according to the human antibody array protocol. Slide arrays were scanned using an Agilent technologies DNA microarray scanner and the analysis was carried out using MetaMorph^®^ software. A representative example of results from the analysis of the hMDM secretome on the RayBiotech L-507 cytokine array is shown in the **Supplementary Material** (**Figure S1**). Internal negative controls were used to establish basal fluorescence and variation across the array. The minimum threshold for a positive fluorescence signal was set at 2.57 standard deviation units above the average background to give a probability of 0.005 that a protein signal would be identified as positive by chance. The linearity of signal detection was verified from internal positive standards. Since signal intensity varied between different arrays, protein expression was normalized to the total fluorescent signal for all proteins on the array. A relative fluorescence value increase of 6500 represented the p<0.005 cutoff compared to negative controls, providing a moderately stringent index for proteins to be considered as actively secreted.

### 2.3 Flow cytometry

Flow cytometry of hMDM at baseline was performed as previously described (60, 61). The hMDM were removed from low adhesion wells in ice cold, calcium-free HBSS and centrifuged for 5 min at 450 x g. Cellular pellets were re-suspended and fixed in a Fluorfix solution (Biolegend 420801) for 20 min at room temperature. Fixed cells were then treated with permeabilization buffer (EBioscience 020-8333-56) and centrifuged for 5 min at 450 x g at 4° C. The wash step was repeated followed by re-suspension in 100 µl of permeabilization buffer plus 5 μl antibody (CD 206 Biolegend 321114; 5 μl CD16 Biolegend 302008 and 5 μl CD163 Biolegend 333607, CD14, Biolegend 301817; CD80, Biolegend 305207; CD86 Biolegend 305420; CD192 Biolegend 335303; CD197 Biolegend 353203, 353205) at room temperature for 20 min. The stained cells were washed three times in cell staining buffer (Biolegend 420201). Flow cytometry was performed on a FACS Calibur (Becton Dickinson, San Jose, CA) using direct immunofluorescence with at least 10,000 events. All cells were gated to remove debris. Three color staining analysis was utilized. Cells were analyzed according to side scatter and receptor bound fluorescence, and data was collected with logarithmic amplifiers. Fluorescence spillover compensation was estimated using single-stained and unstained samples with the Cell Quest software (BD). After collection, data was further analyzed with FlowJo software (TreeStar Inc., Ashland, OR).

### 2.4 Cell treatment

The selective agonists for TLR4 [lipopolysaccharide (LPS); 1 μg/ml] (Cat. #L9641, Lot # 071M4120V, Sigma-Aldrich, Saint Louis, MO, USA), and TLR7 [imiquimod (IMQ); 30 μg/ml] (Cat. #tlrl-imqs, *In vivo*Gen, San Diego, CA, USA) were added to the cultures alone, or together with allopregnanolone (1.0 μM) or 3α,5α-THDOC (1.0 μM) or SGE-516 (1.0 μM) in DMEM (without FBS and antibiotics) 24 h before cell collection. Synthetized allopregnanolone and 3α,5α-THDOC were gifts from Dr. Purdy (62, 63) and SGE-516 was a gift from Sage Therapeutics (54). The ligand and neurosteroid concentrations were selected based on previous findings of maximal effects (33, 34). The effects of the neurosteroids on cells that were not treated with the TLR agonists were studied in parallel.

### 2.5 Protein extraction for immunoblotting and ELISA

Protein extraction and assay were as previously described (33, 34, 64, 65). Cells were lysed with radioimmunoprecipitation (RIPA) buffer (Sigma, Cat. # R0278) supplemented with protease and phosphatase inhibitor cocktails (Sigma, Cat. # P8340 and P0044, respectively). The lysates were sonicated twice for 30 seconds at 25% output power with a Sonicator ultrasonic processor (Misonix, Inc., Farmingdale, NY) and centrifuged (14,000 g; 4°C) for 30 min. Total protein levels were determined by the bicinchoninic acid assay (BCA, Thermo Fisher Scientific, Waltham, MA, USA, Cat.# 23228 and Cat.# 1859078).

### 2.6 Immunoblotting

The proteins (50 μg/lane) were denatured at 95°C (5 min) in 4x Laemmli denaturing buffer (Bio-Rad, Cat. # 1610747) with 10% β-mercaptoethanol and resolved by SDS-polyacrylamide gel (SDS-PAGE) electrophoresis as previously described (33, 34). Briefly, the 10% separation gels (16x18cm) and 3% stacking gels were freshly prepared from acrylamide/bisacrylamide (ratio 29:1) stock solution (Bio-Rad, Cat. # 161-0156) and were polymerized by the addition of 0.025% tetramethylethylenediamine (TEMED; BioRad, Cat. # 1610800EDU) and ammonium persulphate (Bio-Rad, Cat. # 7727540). Electrophoresis was carried out with a current of 25 mA per gel for 4-5 hours. Electrophoretically separated samples were transferred to a polyvinylidene difluoride membrane (PVDF; Bio-Rad Cat. #1620177). Blots were blocked for 2 hrs at room temperature (RT) with 5% blotting-grade blocker (Bio-Rad, Cat. # 1706404) or 5% bovine serum albumin (for phosphorylated primary antibodies) and exposed to primary antibodies overnight (4°C), followed by horseradish peroxidase-labeled secondary antibodies (1 hr, RT). Primary and secondary antibodies were diluted with 5% blotting-grade blocker buffer or 5% BSA (for phosphorylated primary antibodies). Tris-buffered saline with 0.05% Tween-20 (TNT) was used to wash the blots 3 times (10 min each) after incubation with primary and secondary antibodies. Immunoreactive bands were visualized with the PlusECL kit reagents (Perkin Elmer, Waltham, MA, USA, Cat.# NEL105001EA) followed by detection with enhanced chemiluminescence (ImageQuant LAS4000, GE Healthcare, Amersham, UK). Densitometric analysis was conducted using ImageQuant TL v8.1.0.0. Each densitometric measurement was divided by the corresponding β-actin densitometric measurement and the results are expressed as the mean β-actin adjusted densitometric units ± SEM. The primary antibodies, their clonality, host species, dilution and supplier are listed in **SM (Table S1)**. All antibodies were validated by the supplier and by us, as previously described (33, 34, 64, 65). Specific protein detection used full length gels (33, 34, 64, 65). Horseradish peroxidase-labeled secondary antibodies were anti-rabbit (Cat. # 7074, RRID: AB_2099233, Cell Signaling Technology), anti-mouse (Cat# 7076, RRID: AB_330924, Cell Signaling Technology) and anti-goat IgG (Cat# A24452, RRID: AB_2535921, Thermo Fisher Scientific, Waltham, MA, USA).

### 2.7 ELISA

Protein extracts were assayed with ELISA kits (Raybiotech, Norcross, GA, USA) for MCP-1 (Cat. # ELH-MCP1-1), TNF-α (Cat. # ELH-TNFa-CL-1), IL-6 (Cat. # ELH-IL6-CL-1), IL-1β (Cat. # ELH-IL1b-1), IFN-γ (Cat. # ELH-IFNg-CL-1), IL-1ra (Cat. # ELH-IL1ra-CL-1), IL-13 (Cat. # ELH-IL13-1), and TGF-β1 (Cat. # ELH-TGFb1-1) as per the manufacturer’s instructions. Results are expressed as picograms/milligram total protein (pg/mg).

### 2.8 Statistics

Two-way analysis of variance (ANOVA) followed by Tukey’s *post-hoc* test (GraphPad Prism 8.3.1.) was used for the statistical analysis of hMDM cells treated with TLR agonists with/without the neurosteroids. hMDM cultures (n ≥ 12/group) were obtained from 3-5 donors per group. Results are from a total of 27 donors, 11 females and 16 males. P<0.05 was considered statistically significant.

## 3 Results

### 3.1 Characteristics of hMDM

**Figure 1:**
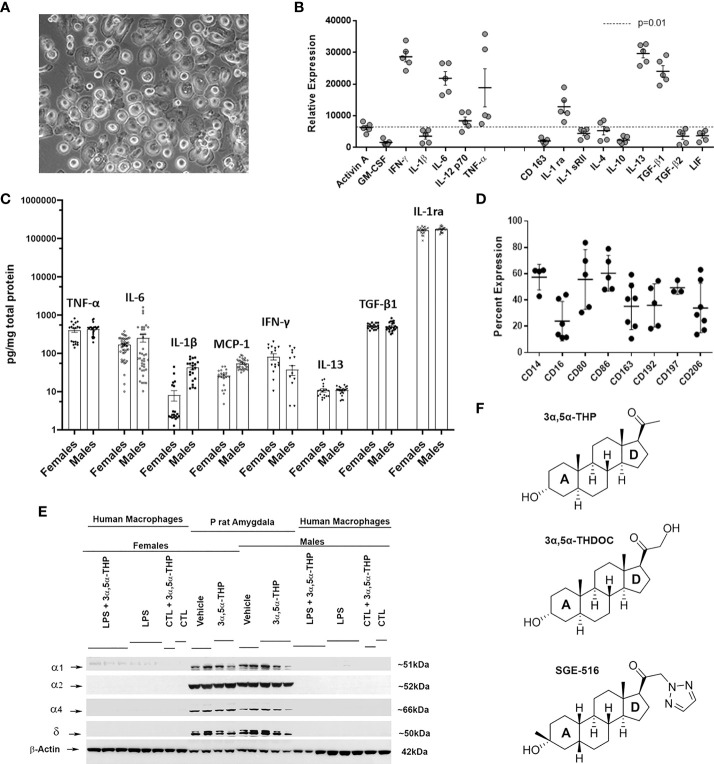
Characteristics of cultured human monocyte-derived macrophages. **(A)** Example of a monolayer of human monocyte-derived macrophages (hMDM) grown on ultralow adhesion plastic. **(B)** Basal hMDM secretome from 5 independent mixed sex cultures detected by RayBiotech human antibody array L-507 illustrating a mixed inflammatory and anti-inflammatory cytokine profile. **(C)** hMDM cell lysates analyzed by ELISA express both inflammatory (TNF-α, IL-6, IL-1β, MCP-1, IFN-γ) and anti-inflammatory (IL-13, TGF-β1, IL-1ra) mediators with no evidence of sex differences at baseline. Each hMDM culture is shown as a single symbol (n ≥ 18), obtained from at least 3 male or 3 female donors. **(D)** hMDM surface markers determined by flow cytometry using direct immunofluorescence in 4-7 independent mixed sex cultures. **(E)** Western blot image shows that hMDM from both male and female donors (n=9 cultures from 3 donors/sex) lack γ-aminobutyric acid type A (GABA_A_) receptor subunits α1, α2, α4, and δ at baseline (CTL) or after treatments with lipopolysaccharide (LPS) and/or allopregnanolone (3α,5α-THP). As a positive control, the amygdala from male and female alcohol preferring P rats intraperitoneally injected with vehicle (45% w/v 2-hydroxypropyl-β-cyclodextrin; 30 min) or allopregnanolone (15 mg/kg; 30 min) was used. **(F)** Chemical structures of endogenous neurosteroids allopregnanolone and 3α,5α-THDOC (tetrahydrodeoxycorticosterone) and the synthetic 1,2,5-triazole analog of the allopregnanolone, SGE-516. 3α,5α-THDOC differs from the allopregnanolone by a C-21-hydroxyl group at the D-ring. SGE-516 differs from the allopregnanolone by a C-3 cis-methyl and cis-hydrogens at C-5 and C-19, and a C-21-1,2,5-triazole group at the D-ring.

The hMDM represent a typical macrophage morphology (**Figure 1A**) as previously described (66). Based on specific expression of cellular surface markers and the secretion of certain cytokines, macrophages can be classified into classically activated, pro-inflammatory macrophages (67–69), and alternatively activated, anti-inflammatory macrophages (68–70). Surface expression of CD14 and CD16 are used to distinguish classical, intermediate, and nonclassical macrophage subsets (71, 72). The basal hMDM secretome was assessed by RayBiotech human antibody array L-507 (**Figure 1B**) and illustrates a mixed profile of the pro-inflammatory (TNF-α, IL-6, IFN-γ), and anti-inflammatory (IL-13, TGF-β1, IL-1ra) mediators (13, 68, 69), in the baseline state. To determine potential sex differences at baseline, hMDM cell lysates obtained from female and male donors were analyzed by ELISA for both pro-inflammatory (TNF-α, IL-6, IL-1β, MCP-1, IFN-γ) and anti-inflammatory (IL-13, TGF-β1, IL-1ra) mediators, with no evidence of sex differences (**Figure 1C**). Surface markers determined by flow cytometry (**Figure 1D**) revealed basal expression of CD14 as well as a mix of markers sensitive to both M1 (CD80, CD86, CD68, CD197) or M2 (CD16, CD163, CD206) polarization and CCL2/MCP-1 chemotaxis (CD192/CCR2). Because previous studies indicate that rodent macrophages and human peripheral blood mononuclear cells express RNAs of some GABA_A_ receptor subunits (73, 74) we examined GABA_A_ receptor subunit α1, α2, α4, and δ protein expression at baseline or after treatments with lipopolysaccharide (LPS) and/or allopregnanolone in hMDM. GABA_A_ receptor subunits investigated were undetectable at baseline or following treatment with LPS and/or allopregnanolone indicating that responses observed in the current studies were not related to GABAergic pharmacology (**Figure 1E**). Positive controls for GABA_A_ receptor subunit expression are shown from the amygdala of male and female P rats intraperitoneally injected with vehicle (45% w/v 2-hydroxypropyl-β-cyclodextrin; 30 min) or allopregnanolone (15 mg/kg; 30 min) (33, 34).

The endogenous neurosteroids allopregnanolone and 3α,5α-THDOC are positive allosteric modulators of the GABA_A_ receptors (36–38, 54) and have similar chemical structures at A, B, and C rings. 3α,5α-THDOC differs from the allopregnanolone by a C-21-hydroxyl group at the D-ring. SGE-516 is a synthetic neuroactive steroid that differs from the allopregnanolone by a C-3 cis-methyl and cis-hydrogens at C-5 and C-19 in the A-ring, and a C-21-1,2,5-triazole group at the D-ring (**Figure 1F**).

### 3.2 Inhibitory effects of allopregnanolone on the TLR4 signaling pathway in male and female hMDM

We have previously shown that allopregnanolone inhibits the activated TLR4 signaling pathway in the mouse macrophage RAW264.7 cell line and the P rat brain (33, 34). Pathway inhibition included blocking of the phosphorylation (activation) of canonical signaling members including cAMP-response element binding protein (CREB), nuclear factor kappa B (NF-kB) p65 and the resulting expression of inflammatory MCP-1, high mobility group box 1 (HMGB1) and TNF-α (33). hMDM were treated with LPS (1 µg/ml; 24h) in the absence or presence of allopregnanolone (1.0 µM), 3α,5α-THDOC (1.0 µM) or SGE-516 (1.0 µM). Cell lysates were assayed for established members of the activated TLR4 pathway, including activated (phosphorylated) pCREB, signal transducer and activator of transcription 1 (pSTAT1), the cytokine TNF-α, and chemokine MCP-1 (21, 33, 75, 76). The ligand and neurosteroid concentrations were selected based on previous findings of maximal effects (33, 34). The effects of allopregnanolone, 3α,5α-THDOC, and SGE-516 on cells that were not treated with the TLR4 agonist LPS were analogously studied.

**Figure 2:**
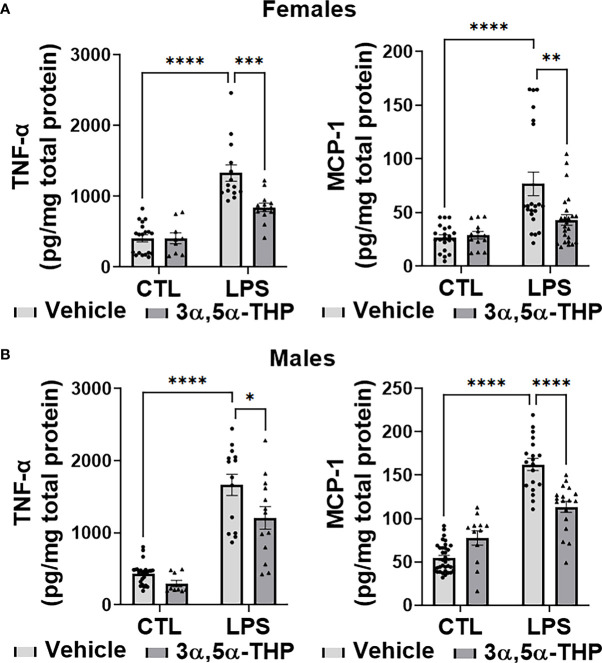
Allopregnanolone (3α,5α-THP) inhibits LPS-induced increases of inflammatory cytokine TNF-α and chemokine MCP-1 in human monocyte-derived macrophages (hMDM) from both female and male donors. Each hMDM culture (n ≥12 cultures from 3-5 female **(A)** or 3-5 male **(B)** donors/grp) is shown as a single symbol. Cells were treated with the TLR4 agonist lipopolysaccharide (LPS) (1 µg/ml; 24h) with or without allopregnanolone (1 µM; 24h). LPS caused a significant increase in the levels of TNF-α (~70% in hMDM from both female and male donors) and MCP-1 (~65% in hMDM from both female and male donors) relative to vehicle control (CTL). The increases of TNF-α were partially inhibited by allopregnanolone (~55% and ~35% inhibition in hMDM from female **(A)** and male **(B)** donors, respectively) (Two-way ANOVA, Tukey’s *post hoc* test: *p < 0.05, ***p < 0.001, ****p < 0.0001). The increases of MCP-1 were completely inhibited by allopregnanolone in hMDM from female **(A)** and by ~40% in hMDM from male **(B)** donors (Two-way ANOVA, Tukey’s *post hoc* test: **p < 0.01, ****p < 0.0001). Allopregnanolone did not change the levels of TNF-α and MCP-1 in hMDM that were not treated with the TLR4 agonist LPS (p>0.05).

LPS caused a significant increase in the levels of TNF-α [~70% in hMDM from both female (F(1,51)=71.40, p<0.0001) and male (F(1,57)=102.5, p<0.0001) donors] and MCP-1 [~65% in hMDM from both female donors (F(1,75)=21.17, p<0.0001) and male donors (F(1,78)=154.8, p<0.0001)], relative to vehicle control (**Figures 2A**, **B**). The increases of TNF-α were partially inhibited by allopregnanolone [~55% and ~35% inhibition in hMDM from female donors (F(1,51)=9.064, p=0.0040) (**Figure 2A**) and male donors (F(1, 57)=8.004, p=0.0064) (**Figure 2B**), respectively]. The LPS-induced elevation of TNF-α and the inhibitory effect of allopregnanolone on TNF-α were similar in hMDM derived from both male donors and female donors (**Figure S2A**). The increases of MCP-1 were completely inhibited by allopregnanolone in hMDM from female donors (F(1,75)=5.039, p=0.0277) (**Figure 2A**) and by ~40% in hMDM from male donors (F(1,78)=5.094, p=0.0268) (**Figure 2B**). The LPS-induced elevation of MCP-1 is higher and the inhibitory effect of 3α,5α-THP on MCP-1 is lesser in hMDM from male donors than female donors (**Figure S2B**). LPS and/or allopregnanolone did not affect TLR4 expression in hMDM from both male and female donors and there are no sex differences in the expression of TLR4 at baseline, after LPS and/or allopregnanolone treatments (**Figure S3**).

**Figure 3:**
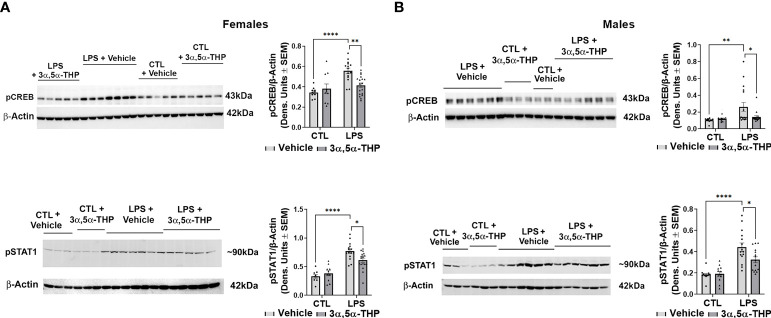
Allopregnanolone (3α,5α-THP) inhibits LPS-induced activation of MyD88-dependent TLR4 pro-inflammatory signal and does not target non-activated TLR4 signal in human monocyte-derived macrophages (hMDM) from both female and male donors. hMDM cultures were treated with LPS (1 µg/ml; 24 h) with or without allopregnanolone (1 µM; 24 h). Representative western blot images and summary data of densitometric scatter dot plots show the levels of pCREB and pSTAT1 in females **(A)** and males **(B)**. Each data point represents an individual hMDM culture (n≥12 cultures from 3 female **(A)** or 3 male **(B)** donors/grp). β-Actin was used as protein loading control. LPS caused a significant increase in the levels of pCREB (~40% and ~60% in MDM from female **(A)** and male **(B)** donors, respectively) and pSTAT1 (~60% in hMDM from both female **(A)** and male **(B)** donors) relative to vehicle control (CTL). The increases of pCREB were completely inhibited by allopregnanolone in hMDM from both female **(A)** and male **(B)** donors. The increases of pSTAT1 were partially inhibited by allopregnanolone (~35% and ~45% inhibition in hMDM from female **(A)** and male **(B)** donors, respectively). Allopregnanolone did not change the levels of pCREB and pSTAT1 in hMDM that were not treated with the TLR4 agonist LPS (A, B), indicating that allopregnanolone specifically targets the activated TLR4 signal. Two-way ANOVA, Tukey’s *post hoc* test: *p < 0.05, **p < 0.01, ****p < 0.0001.

LPS activated the TLR4 pathways through increases in pCREB [~40% and ~60% in hMDM from female (F(1,48)=20.77, p<0.0001) and male (F(1,48)=10.62, p=0.0021) donors, respectively] and pSTAT1 [~60% in hMDM from both female (F(1,51)=81.58, p<0.0001) and male (F(1,49)=48.79, p<0.0001) donors] (**Figure 3**). No effects of LPS (p>0.05) on NF-kB p50 or NF-kB p65 were detected (**Figure S4**). The increases in pCREB were completely inhibited by allopregnanolone in hMDM from both female (F(1,48)=4.428, p=0.0406) (**Figure 3A**) and male (F(1,48)=4.665, p=0.0358) (**Figure 3B**) donors. The increases of pSTAT1 were partially inhibited by allopregnanolone [~35% and ~45% inhibition in hMDM from female (F(1,51)=4.193, p=0.0458) (**Figure 3A**) and male (F(1,49)=4.530, p=0.0384) (**Figure 3B**) donors, respectively]. Consistent with our previous studies (33), allopregnanolone did not change the levels of pCREB, pSTAT1, TNF-α or MCP-1 in hMDM that were not treated with the TLR4 agonist LPS, indicating that allopregnanolone specifically targets the activated TLR4 signal (**Figures 2**, **3**).

### 3.3 3α,5α-THDOC and SGE-516 inhibition of TLR4 pathways in hMDM of female, but not male donors

**Figure 4:**
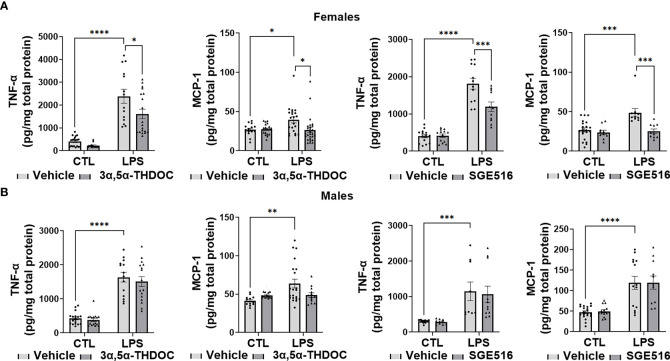
3α,5α-THDOC and SGE-516 inhibit LPS-induced increases of TNF-α and MCP-1 in human monocyte-derived macrophages (hMDM) from female, but not male donors. Each hMDM culture (n≥12 from 3 female **(A)** or 3 male **(B)** donors/grp) is shown as a single symbol. Cells were treated with LPS (1 µg/ml; 24h) with or without 3α,5α-THDOC (1 µM; 24h) or SGE-516 (1 µM; 24h). LPS caused a significant increase in the levels of TNF-α (~80% in hMDM from both female **(A)** and male **(B)** donors) and MCP-1 (~65% in hMDM from both female **(A)** and male **(B)** donors) relative to vehicle control (CTL). The increases of TNF-α were partially inhibited by 3α,5α-THDOC and SGE-516 (~30% and ~35% inhibition, respectively) in hMDM derived from female **(A)** but not male **(B)** donors. The increases of MCP-1 were completely inhibited by 3α,5α-THDOC or SGE-516 in hMDM from female **(A)** but not male **(B)** donors. Two-way ANOVA, Tukey’s *post hoc* test: *p < 0.05, **p < 0.01, ***p < 0.001, ****p < 0.0001.

LPS caused a significant increase in the levels of TNF-α [~80% in hMDM from both female (F(1,63)=88.35, p<0.0001 and F(1,47)=132.7, p<0.0001 for experiments with 3α,5α-THDOC and SGE-516, respectively) and male (F(1,60)=131.3, p<0.0001 and F(1,40)=25.84, p<0.0001 for experiments with 3α,5α-THDOC and SGE-516, respectively) donors] and MCP-1 [~65% in hMDM from both female (F(1,86)=5.067, p=0.0269 and F(1,42)=13.20, p=0.0008 for experiments with 3α,5α-THDOC and SGE-516, respectively) and male (F(1,56)=6.714, p=0.0122 and F(1,55)=48.02, p<0.0001 for experiments with 3α,5α-THDOC and SGE-516, respectively) donors] relative to vehicle control (**Figures 4A**, **B**). The increases of TNF-α were partially inhibited by 3α,5α-THDOC and SGE-516 [~30% and ~35% inhibition, respectively] in hMDM derived from female donors (F(1,63)=7.132, p=0.0096 and F(1,47)=10.12, p=0.0026 for experiments with 3α,5α-THDOC and SGE-516, respectively) (**Figure 4A**), but not male donors (F(1,60)=0.6948, p=0.4078 and F(1,40)=0.09574; p=0.7586 for experiments with 3α,5α-THDOC and SGE-516, respectively) (**Figure 4B**). The increases of MCP-1 were completely inhibited by 3α,5α-THDOC or SGE-516 in hMDM from female donors (F(1,86)=3.981, p=0.0492 and F(1,42)=15.88, p=0.0003 for experiments with 3α,5α-THDOC and SGE-516, respectively) (**Figure 4A**), but not male donors (F(1,56)=0.9187, p=0.3419 and F(1,55)=0.02609, p=0.8723 for experiments with 3α,5α-THDOC and SGE-516, respectively) (**Figure 4B**).

**Figure 5:**
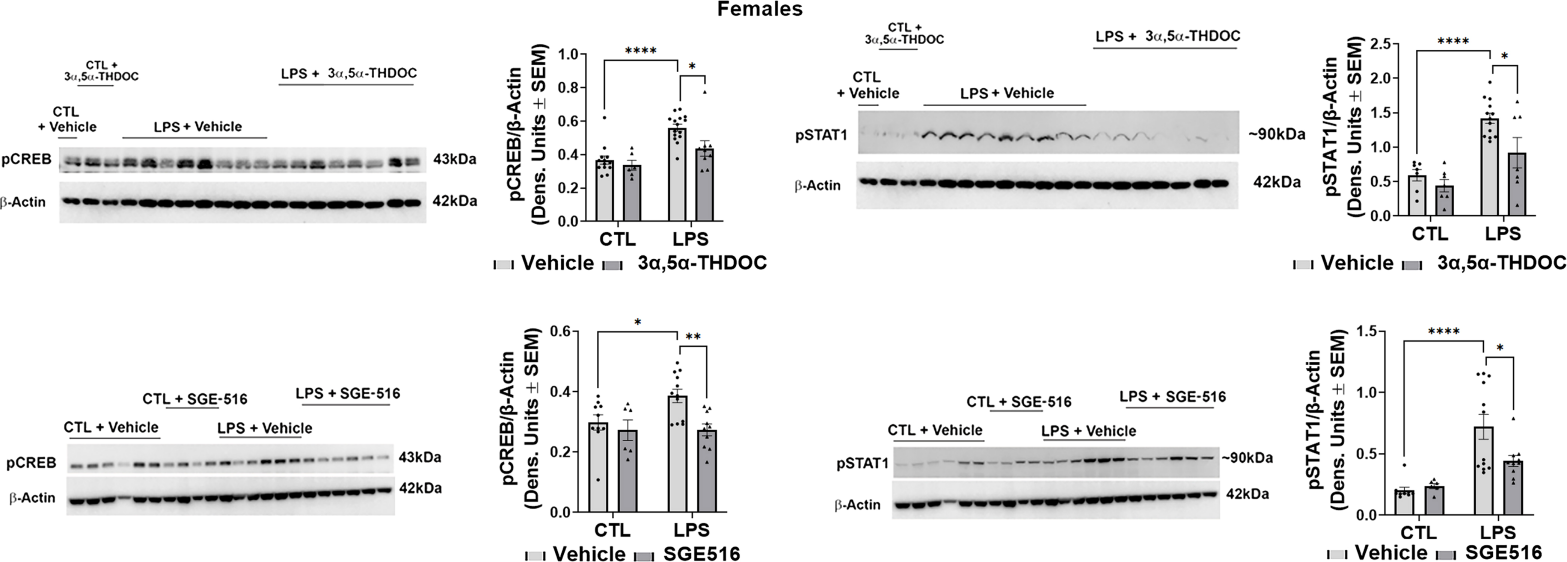
3α,5α-THDOC and SGE-516 inhibit LPS-induced activation of MyD88-dependent TLR4 pro-inflammatory signal in human monocyte-derived macrophages (hMDM) from female donors. hMDM cultures were treated with LPS (1 µg/ml; 24 h) with or without 3α,5α-THDOC (1 µM; 24 h) or SGE-516 (1 µM; 24 h). Representative western blot images and summary data of densitometric scatter dot plots show the levels of pCREB and pSTAT1 in females. Each data point represents an individual hMDM culture (n≥9 from 3 female donors/grp). β-Actin was used as protein loading control. LPS caused a significant increase in the levels of pCREB (~35%) and pSTAT1 (~60%) in hMDM from female donors relative to vehicle control (CTL). The increases of pCREB were completely inhibited by 3α,5α-THDOC and SGE-516. The increases of pSTAT1 were completely inhibited by 3α,5α-THDOC and partially inhibited by SGE-516 (~30%). Two-way ANOVA, Tukey’s *post hoc* test: *p < 0.05, **p < 0.01, ****p < 0.0001.

Since both 3α,5α-THDOC and SGE-516 inhibited LPS-induced increases of TNF-α and MCP-1 in hMDM from female donors, we examined their effects on LPS activation of the TLR4 pathway transcription factors. The levels of pCREB [~35%; F(1,20)=17.13, p=0.0005 and F(1,27)=7.040, p=0.0132 for experiments with 3α,5α-THDOC and SGE-516, respectively] and pSTAT1 [~60%; F(1,30)=28.87, p<0.0001 and F(1,28)=23.92, p<0.0001 for experiments with 3α,5α-THDOC and SGE-516, respectively] were also significantly increased in LPS-treated hMDM from female donors (**Figure 5**). The increases of pCREB were completely inhibited by 3α,5α-THDOC (F(1,20)=4.354, p=0.0499) and SGE-516 (F(1,27)=5.991, p=0.0212). The increases of pSTAT1 were completely inhibited by 3α,5α-THDOC (F(1,30)=7.172, p=0.0119) and partially inhibited by SGE-516 [~30%; F(1,28)=4.732, p=0.0382] (**Figure 5**). Consistent with the allopregnanolone effect, neither 3α,5α-THDOC nor SGE-516 changed the levels of pCREB, pSTAT1, as well as TNF-α or MCP-1 (p>0.05) in hMDM that were not treated with the TLR4 agonist LPS, indicating that both 3α,5α-THDOC and SGE-516 specifically target the activated TLR4 signal (**Figures 4**, **5**).

Collectively, and consistent with our previous studies (33, 34), the data indicate that allopregnanolone inhibits activation of the TLR4 signal and cytokine/chemokine increases in hMDM from both female and male donors. In contrast, 3α,5α-THDOC and SGE-516 inhibit the TLR4 signaling pathway in hMDM from female, but not male donors, indicating a distinct structural requirement for neurosteroids in the inhibition of TLR4 pathways in male macrophages that was not observed in female macrophages.

### 3.4 Inhibitory effects of neurosteroids on the TLR7 signaling pathway are dependent upon sex in hMDM

We have previously shown that allopregnanolone inhibits the activated TLR7 signaling pathway in the mouse macrophage RAW264.7 cell line and the brain of female P rats (34). To examine whether allopregnanolone as well as 3α,5α-THDOC and SGE-516 also inhibit activation of TLR7 signal and pathway members in hMDM, cells were treated with the TLR7 agonist imiquimod (IMQ) (30 µg/ml; 24 hrs) in the absence or presence of allopregnanolone (1.0 µM), 3α,5α-THDOC (1.0 µM) or SGE-516 (1.0 µM). Cell lysates were assayed for established members of the activated TLR7 pathway, including the TLR7-associated activated (phosphorylated) transcription factor interferon regulatory factor 7 (pIRF7), pSTAT1, as well as cytokines IL-6 and IL-1β (19, 34, 77–79). The effect of allopregnanolone, 3α,5α-THDOC or SGE-516 on cells that were not treated with the TLR7 agonist IMQ was analogously studied.

#### 3.4.1 Allopregnanolone, 3α,5α-THDOC and SGE-516 inhibition of TLR7 pathways in hMDM of female, but not male donors

**Figure 6:**
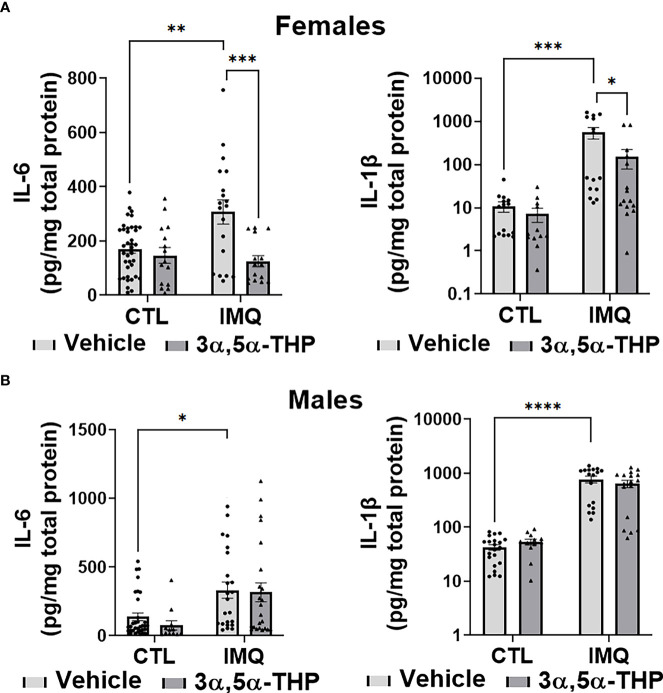
Allopregnanolone (3α,5α-THP) inhibits imiquimod-induced increases of inflammatory cytokines IL-6 and IL-1β in human monocyte-derived macrophages (hMDM) from female but not male donors. Each hMDM culture (n≥12 cultures from 3-5 female **(A)** or 3-5 male **(B)** donors/grp) is shown as a single symbol. Cells were treated with TLR7 agonist imiquimod (IMQ; 30 µg/ml) with or without allopregnanolone (1 µM). Cells were harvested at 24 h after treatment initiation and examined for the expression of inflammatory cytokines IL-6 and IL-1β. IMQ caused a significant increase in the levels of IL-6 (~45% and ~60% in hMDM from female **(A)** and male **(B)** donors, respectively) and IL-1β (~95% in hMDM from both female **(A)** and male **(B)** donors), relative to vehicle control (CTL) (Two-way ANOVA, Tukey’s *post hoc* test: *p < 0.05, **p < 0.01, ***p < 0.001, ****p < 0.0001). The increases of IL-6 and IL-1β, were completely inhibited by allopregnanolone in hMDM from female donors (Two-way ANOVA, Tukey’s *post hoc* test: *p < 0.05, ***p < 0.001) but not male donors (p > 0.05). Allopregnanolone did not change the levels of IL-6 and IL-1β (p>0.05) in hMDM that were not treated with the TLR7 agonist IMQ.

**Figure 7:**
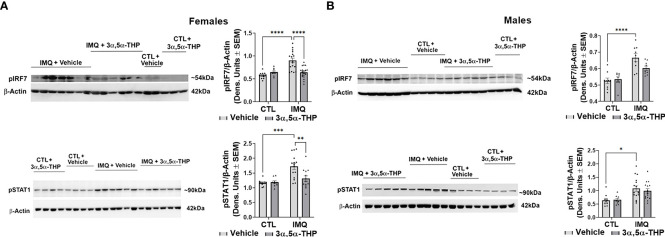
Allopregnanolone (3α,5α-THP) inhibits imiquimod-induced activation of MyD88-dependent TLR7 pro-inflammatory signal in human monocyte-derived macrophages (hMDM) from female but not male donors. hMDM were treated with TLR7 agonist imiquimod (IMQ; 30 µg/ml) with or without allopregnanolone (1 µM). Cells were harvested at 24 h after treatment initiation and examined for the expression of MyD88-dependent activated (phosphorylated) interferon regulatory factor 7 (pIRF7) and transcription factor pSTAT1. Representative western blot images and summary data of densitometric scatter dot plots show the levels of pIRF7 and pSTAT1 in females **(A)** and males **(B)**. Each data point represents an individual hMDM culture (n≥12 cultures from 3 female **(A)** or 3 male **(B)** donors/grp). β-Actin was used as protein loading control. IMQ caused a significant increase in the levels of pIRF7 (~35% and ~25% in hMDM from female **(A)** and male **(B)** donors, respectively) and pSTAT1 (~35% and ~40% in hMDM from female **(A)** and male **(B)** donors, respectively) relative to vehicle control (CTL) (Two-way ANOVA, Tukey’s *post hoc* test: *p < 0.05, ***p < 0.001, ****p < 0.0001). The increases of pIRF7 and pSTAT1 were completely inhibited by allopregnanolone in hMDM from female donors (Two-way ANOVA, Tukey’s *post hoc* test: **p < 0.01, ****p < 0.0001) but not male donors (Two-way ANOVA, Tukey’s *post hoc* test, p>0.05). Allopregnanolone did not change the levels of pIRF7 and pSTAT1 (p>0.05) in hMDM that were not treated with the TLR7 agonist IMQ indicating that allopregnanolone specifically targets the activated TLR7 signal.

**Figure 8:**
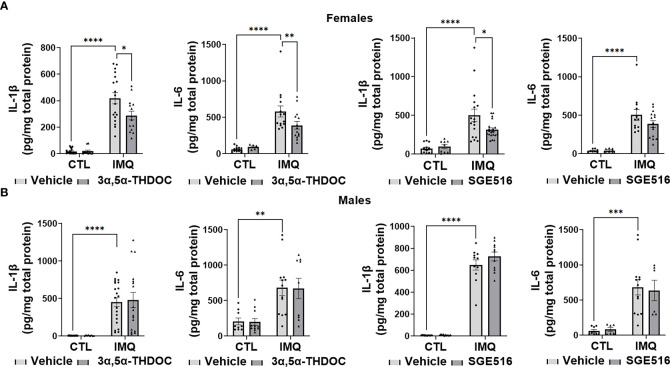
3α,5α-THDOC partially inhibits imiquimod-induced increases of IL-1β and IL-6 and SGE-516 partially inhibits IL-1β in human monocyte-derived macrophages (hMDM) from female but not male donors. Each hMDM culture (n≥12 from 3 female **(A)** or 3 male **(B)** donors/grp) is shown as a single symbol. Cells were treated with imiquimod (IMQ) (30 µg/ml; 24h) with or without 3α,5α-THDOC (1 µM; 24h) or SGE-516 (1 µM; 24h). IMQ caused a significant increase in the levels of IL-1β (~95% in hMDM from both female **(A)** and male **(B)** donors) and IL-6 (~90% and ~80% in MDM from female **(A)** and male **(B)** donors, respectively) relative to vehicle control (CTL). The increases of IL-1β were partially inhibited by 3α,5α-THDOC and SGE-516 (~30% and ~35% inhibition, respectively) in hMDM derived from female **(A)** but not male **(B)** donors. The increases of IL-6 were partially inhibited by 3α,5α-THDOC in hMDM from female (~30% inhibition) **(A)** but not male **(B)** (p>0.05) donors and were not inhibited by SGE-516 in hMDM from both female **(A)** and male **(B)** donors (p>0.05). Two-way ANOVA, Tukey’s *post hoc* test: *p < 0.05, **p < 0.01, ***p < 0.001, ****p < 0.0001.

IMQ caused a significant increase in the levels of IL-6 [~45-90% and 60-80% in hMDM from female donors (F(1,82)=4.648, p=0.0340; F(1,42)=49.41, p<0.0001 and F(1,38)=56.34, p<0.0001 for experiments with allopregnanolone, 3α,5α-THDOC and SGE-516, respectively) (**Figures 6A**, **8A**) and male donors (F(1,88)=15.55, p=0.0002; F(1,32)=21.25, p<0.0001 and F(1,22)=33.00, p<0.0001 for experiments with allopregnanolone, 3α,5α-THDOC and SGE-516, respectively)] (**Figures 6B**, **8B**). Likewise, IMQ caused a significant increase in the levels of IL-1β [~95% in hMDM from both female donors (F(1,44)=8.416, p=0.0058; F(1,58)=127.0, p<0.0001 and F(1,51)=47.46, p<0.0001 for experiments with allopregnanolone, 3α,5α-THDOC and SGE-516, respectively) (**Figures 6A**, **8A**) and male donors (F(1,63)=73.33, p<0.0001; F(1,59)=40.79, p<0.0001 and F(1,43)=553.6, p<0.0001 for experiments with allopregnanolone, 3α,5α-THDOC and SGE-516, respectively)] (**Figures 6B**, **8B**). IMQ activated the TLR7 pathways through increases in pIRF7 [~35% and ~25% in hMDM from female donors (F(1,52)=18.15, p<0.0001) (**Figure 7A**) and male donors (F(1,24)=18.45, p=0.0002)] (**Figure 7B**) and pSTAT1 [~35% and ~40% in hMDM from female donors (F(1,42)=13.64, p=0.0006) (**Figure 7A**) and male donors (F(1,45)=15.61, p=0.0003)] (**Figure 7B**).

The increases of IL-6 were completely inhibited by allopregnanolone in hMDM from female donors (F(1,82)=12.45, p=0.0007) (**Figure 6A**), but not male donors (F(1,88)=0.5031, p=0.4800) (**Figure 6B**). The increases of IL-1β were completely inhibited by allopregnanolone in hMDM from female donors (F(1,44)=4.148, p=0.0477) (**Figure 6A**), but not male donors (F(1,63)=0.6435, p=0.4254) (**Figure 6B**). The increases of pIRF7 were completely inhibited by allopregnanolone in hMDM from female donors (F(1,52)=6.619, p=0.0130) (**Figure 7A**), but not male donors (F(1,24)=0.1615, p=0.6913) (**Figure 7B**). The increases of pSTAT1 were completely inhibited by allopregnanolone in hMDM from female donors (F(1,42)=4.489, p=0.0401) (**Figure 7A**), but not male donors (F(1,45)=0.2987, p=0.5874) (**Figure 7B**). IMQ and/or allopregnanolone did not affect TLR7 expression in hMDM from both male and female donors and there are no sex differences in the expression of TLR7 at baseline, after IMQ and/or allopregnanolone treatments (**Figure S5**).

Consistent with the inhibitory effect of allopregnanolone, 3α,5α-THDOC and SGE-516, albeit to a lesser extent, also inhibited IMQ-induced cytokine increases in hMDM derived from female donors, but not male donors (**Figures 8A, B**). The increases of IL-1β were partially inhibited by 3α,5α-THDOC and SGE-516 [~30% and ~35% inhibition, respectively] in hMDM derived from female donors (F(1,58)=4.702, p=0.0343 and F(1,51)=4.744, p=0.0340, for experiments with 3α,5α-THDOC and SGE-516, respectively) (**Figure 8A**), but not male donors (F(1,59)=0.04079, p=0.8406 and F(1,43)=1.924, p=0.1725 for experiments with 3α,5α-THDOC and SGE-516, respectively) (**Figure 8B**). The increases of IL-6 were partially inhibited by 3α,5α-THDOC [~30% inhibition] in hMDM derived from female donors (F(1,42)=4.202, p=0.0467) (**Figure 8A**), but not male donors (F(1,32)=0.006, p=0.9396) (**Figure 8B**), but were not inhibited by SGE-516 in hMDM from either female donors (F(1,38)=1.094, p=0.3022) (**Figure 8A**) or male donors (F(1,22)=0.011, p=0.9174) (**Figure 8B**).

Consistent with our previous studies (34), in the absence of the TLR7 agonist IMQ, allopregnanolone did not change the levels of pIRF7, pSTAT1, IL-6 or IL-1β and 3α,5α-THDOC and SGE-516 did not change the levels of IL-6 or IL-1β in hMDM, indicating that the neurosteroids specifically target the activated TLR7 signal (**Figures 6**–**8**).

Collectively, these results extend our previous studies in the mouse RAW264.7 macrophage cell line (34) to primary human macrophages, indicating that allopregnanolone inhibits activation of the TLR7 signal and cytokine increases. However, we show here that its effects are selective for hMDM from female donors. Correspondingly, although with lesser efficacy, 3α,5α-THDOC and SGE-516 also inhibit the TLR7 signaling pathway in hMDM from female, but not male donors, indicating sex specificity favoring females in the inhibition of TLR7 pathways by all the neurosteroids tested.

## 4 Discussion

The current findings indicate that allopregnanolone inhibits activation of the TLR4 and TLR7 signals and cytokine/chemokine increases in hMDM. However, we show here that its effects in hMDM are distinct from RAW264.7 cells (29), as the inhibition of TLR7 pathways are selective for female donors. We further observe that two steroids with C-21 structural modifications, 3α,5α-THDOC and SGE-516 inhibit the TLR4 pathway in hMDM from female, but not male donors, indicating different structural specificity for neurosteroid inhibition of TLR4 pathways in male vs. female donors. We also demonstrate that allopregnanolone, 3α,5α-THDOC and SGE-516 inhibit the activated TLR signaling pathways in hMDM, but they have no effect on the unstimulated pathways.

The TLR4 agonist LPS increased multiple markers of TLR4 activation, including the phosphorylated (activated) transcription factors CREB, STAT1, as well as TNF-α and MCP-1, in hMDM. All these effects were inhibited by allopregnanolone in hMDM from both male and female donors. In contrast 3α,5α-THDOC and SGE-516 inhibit these TLR4 pathway members in hMDM from female, but not male donors, indicating different structural requirements of neurosteroids for inhibiting TLR4 signaling between sexes. We previously demonstrated that allopregnanolone inhibits TLR4 binding to both MyD88 and MD2 to prevent TLR4 pathway activation and the production of MCP-1, HMGB1 and TNF-α in RAW264.7 cells (33). The present results suggest that TLR4 pathway activation mechanisms may differ in female vs. male hMDM, such that 3α,5α-THDOC and SGE-516 are unable to block their activation in males. This might involve sex differences in TLR4 adaptor protein expression that alter the structural requirements for inhibition of pathway activation. Further studies are needed to elucidate the mechanism of this sex difference.

The TLR7 agonist IMQ increased markers of TLR7 activation, phosphorylated (activated) transcription factors STAT1 and IRF7, as well as IL-6 and IL-1β, in hMDM. All these effects were inhibited by allopregnanolone in hMDM from female, but not male donors. Correspondingly, 3α,5α-THDOC inhibits IL-6 and IL-1β and SGE-516 inhibits IL-1β in hMDM from female, but not male donors. The mechanism of the sex specificity in TLR7 signal inhibition by neurosteroids in hMDM is unknown and additional studies will be required for elucidation. Since allopregnanolone inhibits the TLR7 interaction with MyD88 (34), it is likely that other TLR7 adaptor proteins as well as sex hormone receptors, chaperons, or proteins that enhance or suppress TLR7 signals may be involved.

Even though the endogenous neurosteroids allopregnanolone and 3α,5α-THDOC, and synthetic compound SGE-516 are all allosteric modulators of GABA_A_ receptors (36–38, 54), the present data suggests that GABAergic activity may not be necessary or sufficient for TLR4 inhibition in hMDM, similar to our previous report in RAW264.7 cells (33). We previously found that allopregnanolone and pregnenolone (which lacks GABAergic actions) both completely inhibit TLR4 pathway activation and the production of MCP-1, HMGB1 and TNF-α in RAW264.7 cells (33). These two steroids have distinct A ring properties, with identical D ring structure, suggesting structural specificity at ring D for inhibition of TLR4 signaling in RAW264.7 cells (33). Since the structures of both 3α,5α-THDOC and SGE-516 differ from the allopregnanolone by additional C-21-hydroxyl and C-21-1,2,5-triazole groups, respectively (54, 62, 80), modifications such as these at the D-ring could be detrimental for neurosteroid inhibition of TLR activation in hMDM from male donors as well as RAW264.7 cells. Further additional studies with a greater number of structural modifications are needed to adequately assess the structural requirements for TLR inhibition. Moreover, the effects of these structural modifications on neurosteroid inhibition of TLRs in the brain also remain unstudied.

Even though these data indicate that there are no sex differences in the expression of both TLR4 and TLR7 at baseline, or pathway activation by agonists, we observed differential inhibitory effects of neurosteroids in female vs. male macrophages. Numerous sex differences in innate and adaptive immunity have been identified in various studies (81–85). Sex differences in immune function can be attributed in many cases to regulatory effects of gonadal hormones *in vivo* (86). Other sex differences include reduced sensitivity to the effects of LPS, greater phagocytic activity, production of more anti-inflammatory prostanoids, and more efficient antigen presentation in female macrophages (86, 87). The present data indicate that female macrophages had reduced sensitivity to LPS, as defined by the level of MCP-1, compared to male macrophages (**Figure S1**). This factor may explain the greater inhibition of LPS-induced elevation of MCP-1 by allopregnanolone in female macrophages when compared with male macrophages. Thus, neurosteroid regulation may represent an additional mechanism for sex dependent immune regulation.

We previously showed that allopregnanolone inhibits TLR4 binding to MD-2 and MyD88, and TLR7 binding to MyD88 in RAW264.7 cells (33, 34). However, the exact mechanism of allopregnanolone inhibition remains unclear. Allopregnanolone and pregnenolone have also been shown to enhance the degradation of bound adaptor proteins, which promote ubiquitination and degradation of the toll/interleukin-1 receptor domain-containing adapter protein and TLR2 in HEK293T cells (88). Further studies are needed to identify the protein-protein interactions that are inhibited by neurosteroids and enable kinetic analysis.

Pro-inflammatory signaling through TLRs plays a major role in the detrimental activities of macrophages (15). The TLR4-specific ligand LPS, causes receptor oligomerization with multiple adaptor proteins at the cell membrane, inducing a cascade of protein-protein interactions that produce proinflammatory cytokines and chemokines (16, 19, 75, 89–91). TLR7 is located on the endosome and recognizes single-stranded RNA molecules (ssRNAs) (92, 93) and imidazoquinoline derivatives such as IMQ, which directly bind TLR7, induce its dimer formation, MyD88 binding and subsequent production of proinflammatory cytokines (16, 94, 95). The ability of allopregnanolone to simultaneously inhibit both TLR4 and TLR7 pathway activation could offer much needed control of inflammatory signaling and may offer a unique approach to the treatment of inflammatory conditions. Thus, the demonstration of TLR4 and TLR7 inhibition by various neurosteroids in hMDM has clear therapeutic relevance.

Macrophages promote neuroinflammation following traumatic brain injury (96), in multiple sclerosis (97, 98), ischemic stroke and intracerebral hemorrhage (98), and contribute to systemic inflammation in many other disease states (10). In addition, systemic inflammation is thought to contribute to the development and progression of neurodegenerative diseases (99). Moreover, various proinflammatory cytokines and chemokines derived from TLR4 and TLR7 activation are now recognized as potential markers of psychiatric conditions, including depression (24, 25), postpartum depression (100, 101), post-traumatic stress disorders (102–104) and alcohol use disorders (26, 27, 105). Thus, it seems plausible that the ability of allopregnanolone to inhibit both TLR4 and TLR7 interactions with MyD88 and their pathways contribute to its therapeutic actions in the treatment of post-partum depression (43) and post-traumatic stress disorders (106) and may have beneficial actions in other inflammatory conditions.

In conclusion, the present results show that endogenous neuroactive steroids regulate both TLR4 and TLR7 activation in cultured human macrophages, but with specificity for female donors. Because these steroids are present in circulation of both males and females, but are elevated during the luteal phase of the menstrual cycle as well as during pregnancy in women (39), they may play an integral role in the modulation of pro-inflammatory signaling across the lifespan and contribute to protection from both systemic and neuroinflammatory disease. Further work is needed to establish the contribution of their anti-inflammatory effects in the treatment of post-partum depression as well as other inflammatory conditions.

## Data availability statement

The original contributions presented in the study are included in the article/**Supplementary Material**. Further inquiries can be directed to the corresponding author.

## Ethics statement

The studies involving human participants were reviewed and approved by Human buffy coat leukocytes were obtained from healthy donors at the New York Blood Center (http://nybloodcenter.org/), a non-profit organization for the collection and distribution of blood for clinical and research purposes. No personal identifiers were sent with the shipment. The patients/participants provided their written informed consent to participate in this study.

## Author contributions

IB, LA and ALM conceived project; IB, KW and RBM conducted experiments and analyzed data, ALM, RBM and BC provided funding, IB, RBM and ALM drafted manuscript, all authors contributed to the experimental design and editing of manuscript.

## Funding

This work was supported by NIH grant R01-AA024095, Sage Therapeutics Inc., and the Bowles Center for Alcohol Studies at UNC School of Medicine to ALM and R01-NS108808 to RBM.

## Acknowledgements

We would like to thank Ashley Suchy for her work in support of these studies. We dedicate this work to Dr. Laure Aurelian who passed away in May 2021 in the middle of these studies. Her knowledge, inspiration and insight were instrumental in this work and in our efforts.

## Conflict of interest

ALM, LA and IB hold a provisional patent on the anti-inflammatory actions of allopregnanolone and related steroids on Toll-like receptor pathways in the immune system and brain. BC was employed by Sage Therapeutics and the study received partial funding from Sage Therapeutics. Sage Therapeutics contributed the compound SGE-516 for these studies, but had no other involvement in the study.

The remaining authors declare that the research was conducted in the absence of any commercial or financial relationships that could be construed as a potential conflict of interest.

## Publisher’s note

All claims expressed in this article are solely those of the authors and do not necessarily represent those of their affiliated organizations, or those of the publisher, the editors and the reviewers. Any product that may be evaluated in this article, or claim that may be made by its manufacturer, is not guaranteed or endorsed by the publisher.
