# Supplementary material for: Inhibition of human macrophage activation via pregnane neurosteroid interactions with toll-like receptors: Sex differences and structural requirements
Source: Front Immunol. 2022 Jul 29;13:940095. doi: 10.3389/fimmu.2022.940095 (PMC9373802; doi:10.3389/fimmu.2022.940095)
Supplement: Supplementary file 1 [file DataSheet_1.pdf]

Supplementary Material

1. Supplementary Tables

The primary antibodies, their clonality, host species, dilution and supplier are listed in Table S1.

Table S1. Clonality, host species and dilutions of primary antibodies used in immunoblotting

Target	Catalog no	Commercial supplier	Clonality	Host	Dilution
pIRF7 (Ser471/472)	5184	Cell Signaling Technology, Danvers, MA, USA	Polyclonal	Rabbit	1:1000
pCREB (Ser133)	9198	Cell Signaling Technology, Danvers, MA, USA	Monoclonal	Rabbit	1:1000
pNF-kB p65 (Ser536)	3036	Cell Signaling Technology, Danvers, MA, USA	Monoclonal	Mouse	1:1000
pNF-kB p50 (Ser337)	sc-271908	Santa Cruz Biotechnology, Santa Cruz, CA, USA	Monoclonal	Mouse	1:200
pSTAT1 (Ser727)	9177	Cell Signaling Technology, Danvers, MA, USA	Polyclonal	Rabbit	1:1000
GABA A receptor α1	AB5592	Millipore, Temecula, CA, USA	Polyclonal	Rabbit	1:2000
GABA A receptor α2	LS-C413660	LifeSpan BioSciences, Seattle, WA, USA	Polyclonal	Rabbit	1:2000
GABA A receptor α4	ab117080	Abcam, Boston, MA, USA	Polyclonal	Goat	1:700
GABA A receptor δ	NB300-200	Novus Biologicals, Centennial, CO, USA	Polyclonal	Rabbit	1:1000
TLR4	sc-293072	Santa Cruz Biotechnology, Santa Cruz, CA, USA	Monoclonal	Mouse	1:1000
TLR7	5632	Cell Signaling Technology, Danvers, MA, USA	Monoclonal	Rabbit	1:500
β-Actin	66009-1-Ig	Proteintech Group, Rosemont, IL, USA	Monoclonal	Mouse	1:3000

2. Supplementary Results

A representative example of results from the analysis of the human monocyte-derived macrophage (hMDM) secretome on the RayBiotech L-507 cytokine array (RayBiotech Life, Inc., Peachtree, GA) is shown in Fig. S1.

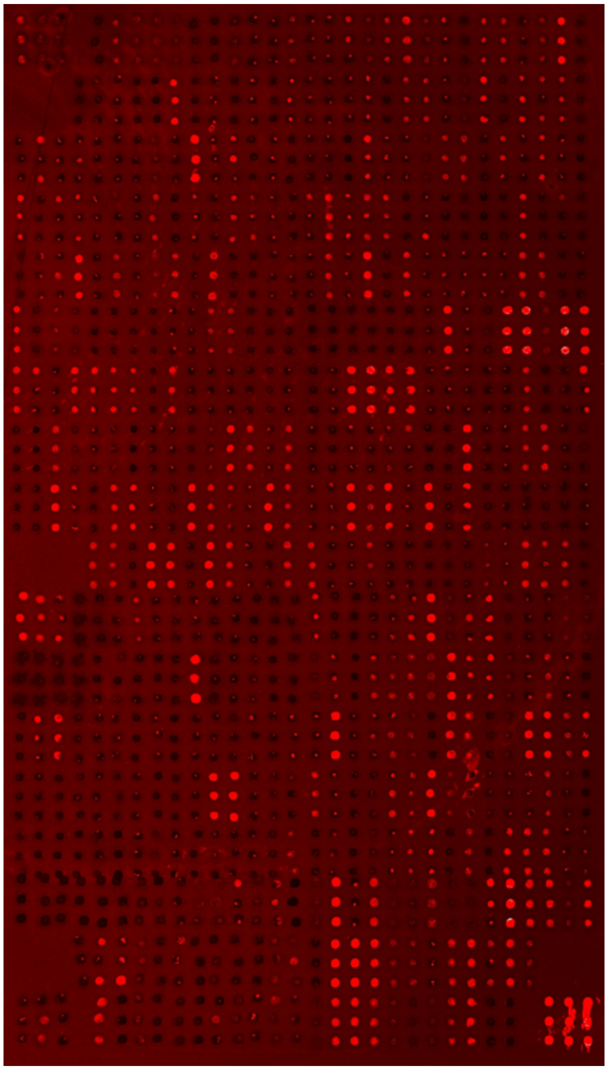

Supplementary Figure S1. Example of results from the analysis of the hMDM secretome on the RayBiotech L-507 cytokine array. Medium was collected from hMDM cultured overnight in serum free DMEM and centrifuged at 1000 x g. The cell free medium was added to a RayBiotech human antibody array L-507 and processed according to the RayBiotech human antibody array protocol. Slide arrays were scanned using an Agilent technologies DNA microarray scanner.

Female macrophages had a reduced sensitivity to lipopolysaccharide (LPS) as defined by the level of MCP-1 compared to male macrophages. This factor may explain the greater inhibition of LPS-induced elevation of MCP-1 by allopregnanolone (3α,5α-THP) in female macrophages when compared with male macrophages (Fig. S2).

A

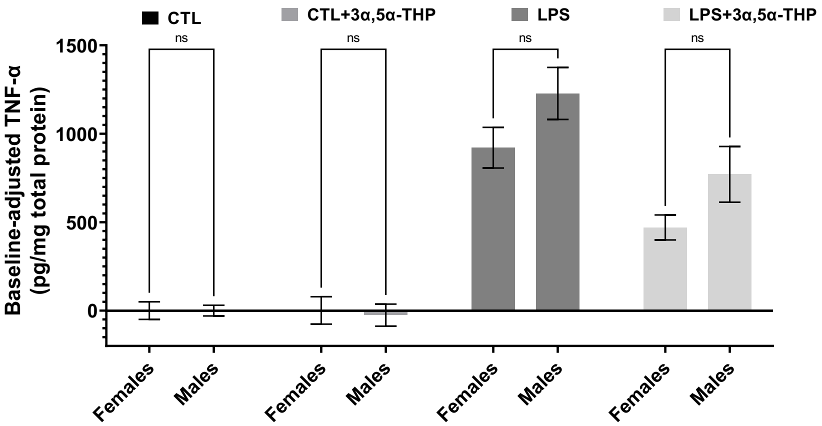

B

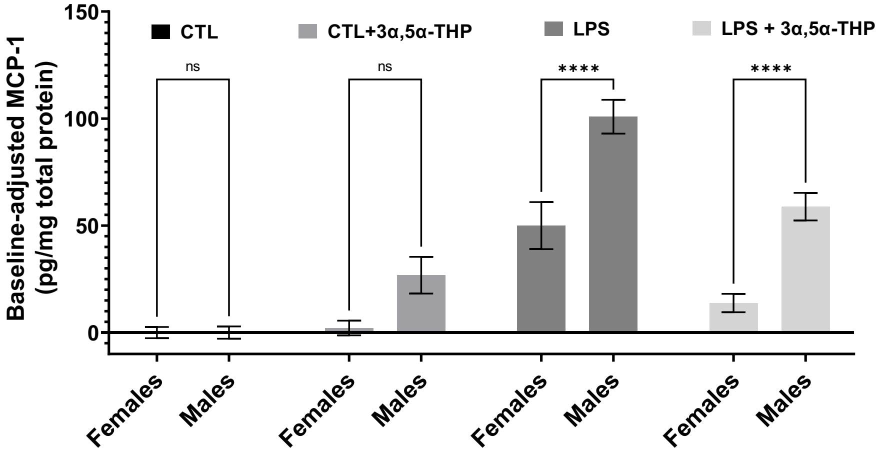

Supplementary Figure S2. Human monocyte-derived macrophage (hMDM) cultures (n≥12 cultures from 3 female or 3 male donors/grp) were treated with LPS (1 µg/ml) with or without allopregnanolone (3α,5α-THP; 1 µM; 24h). **A:** The LPS-induced elevations of TNF-α and the inhibitory effect of 3α,5α-THP on TNF-α were similar in hMDM derived from male donors and female donors (Three-way ANOVA: F(1,100)=3.469, p=0.0655). **B:** The LPS-induced elevations of MCP-1 is higher and the inhibitory effect of 3α,5α-THP on MCP-1 is lesser in hMDM from male donors than female donors (Three-way ANOVA: F(1,155)=42.82, ****p<0.0001). ns – statistically not significant.

Lipopolysaccharide (LPS) and/or allopregnanolone (3α,5α-THP) did not affect TLR4 expression in hMDM from both male and female donors and there are no sex differences in the expression of TLR4 at baseline, after LPS and/or allopregnanolone treatments (Fig. S3).

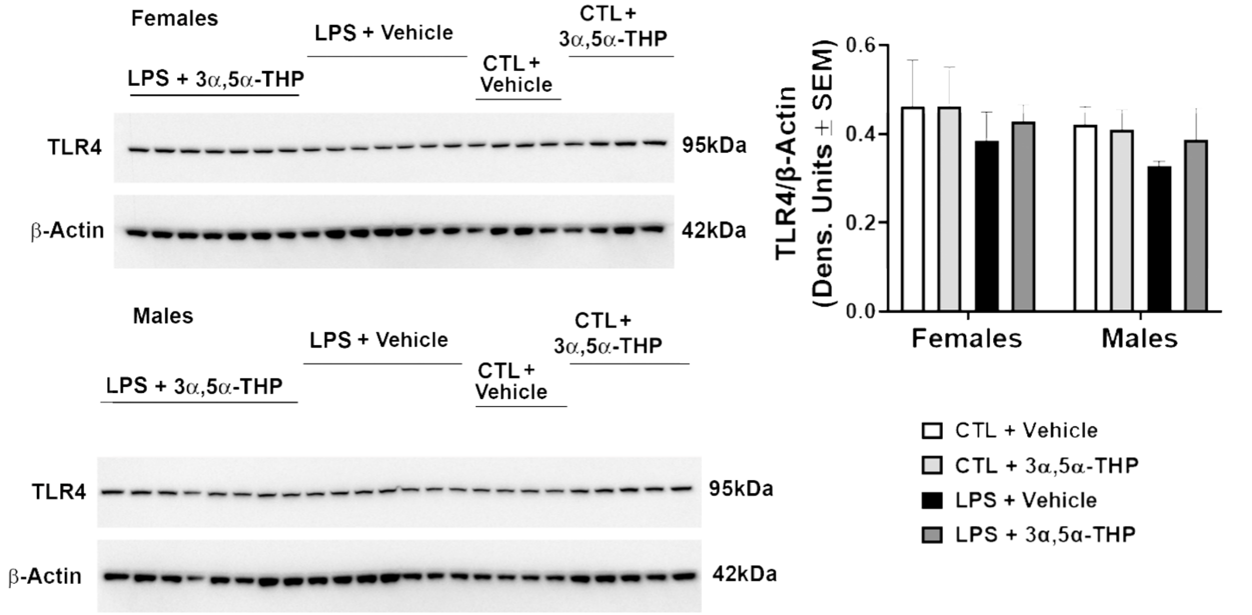

Supplementary Figure S3. Lipopolysaccharide (LPS) and/or allopregnanolone (3α,5α-THP) did not affect TLR4 expression in human monocyte-derived macrophages (hMDM). hMDM cultures (n≥9 cultures from 3 female or 3 male donors/grp) were treated with LPS (1 µg/ml) with or without allopregnanolone (3α,5α-THP; 1 µM). Cells were harvested at 24 h after treatment initiation and examined for the expression of TLR4. Treatments with lipopolysaccharide (LPS) and/or allopregnanolone (3α,5α-THP) did not change TLR4 expression in hMDM from both female and male donors when compared with vehicle control (CTL). There were no differences in the levels of TLR4 between females and males at baseline or after treatments with LPS with or without allopregnanolone (3α,5α-THP). Three-way ANOVA, Tukey's post hoc test, $p>0.05$.

No effects of lipopolysaccharide (LPS; $p>0.05$) on phosphorylated NF-kB p65 (pNF-kB p65) or phosphorylated NF-kB p50 (pNF-kB p50) were detected (Fig. S4).

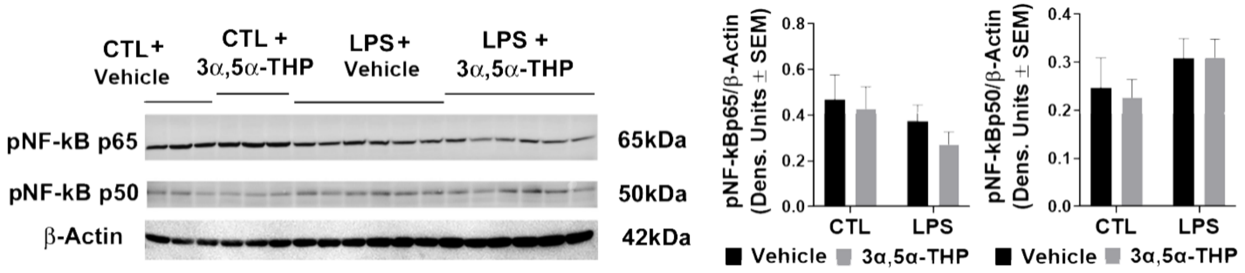

Supplementary Figure S4. Lipopolysaccharide (LPS) and/or allopregnanolone (3α,5α-THP) did not affect the expression of both phosphorylated NF-kB p65 (pNF-kBp65) and phosphorylated NF-kB p50 (pNF-kBp50) in human monocyte-derived macrophages (hMDM). hMDM cultures (n≥9 cultures from 3 female or 3 male donors/grp)) were treated with LPS (1 µg/ml) with or without allopregnanolone (3α,5α-THP; 1 µM). Cells were harvested at 24 h after treatment initiation and examined for the expression of pNF-kB p65 and pNF-kB p50. Treatments with lipopolysaccharide (LPS) and/or allopregnanolone (3α,5α-THP) did not change pNF-kBp 65 or pNF-kBp 50 expression in hMDM from female donors when compared with vehicle control (CTL). The similar results were obtained for hMDM derived from male donors (data not shown). Two-way ANOVA, Tukey's post hoc test, $p>0.05$.

Imiquimod (IMQ) and/or allopregnanolone (3α,5α-THP) did not affect TLR7 expression in hMDM from both male and female donors and there are no sex differences in the expression of TLR7 at baseline, after IMQ and/or allopregnanolone treatments (Fig. S5).

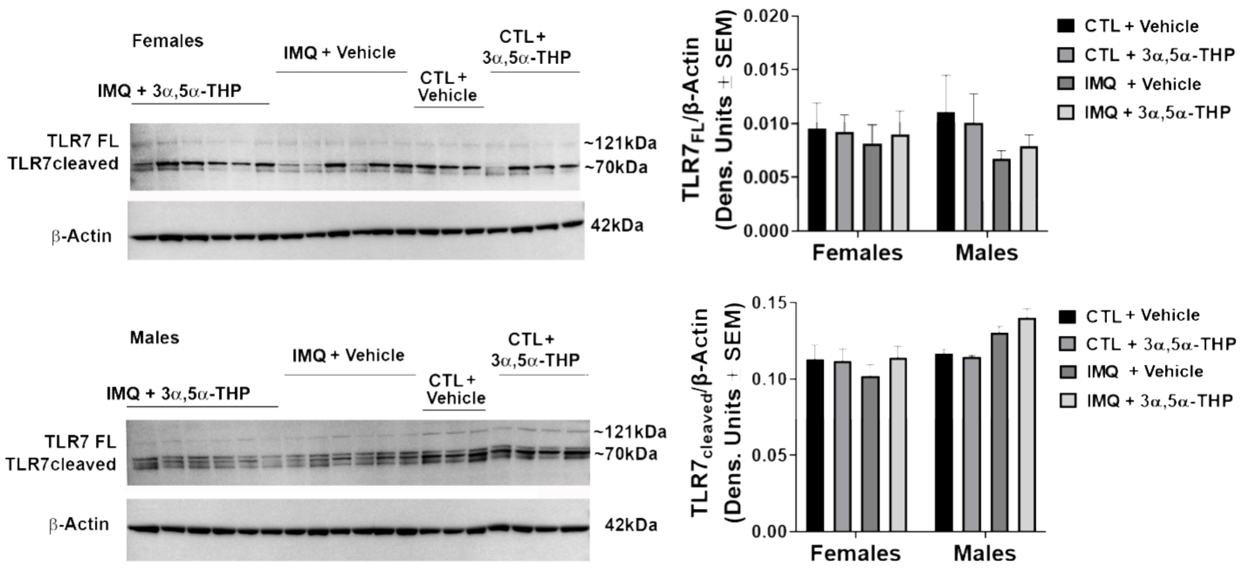

Supplementary Figure S5. Imiquimod (IMQ) and/or allopregnanolone (3α,5α-THP) did not affect TLR7 expression in human monocyte-derived macrophages (hMDM). hMDM cultures (n≥9 cultures from 3 female or 3 male donors/grp) were treated with IMQ (30 μg/ml) with or without allopregnanolone (3α,5α-THP; 1 μM). Cells were harvested at 24 h after treatment initiation and examined for the expression of both full length (FL; ~121 kDa) and cleaved fragment (~70 kDa) of TLR7. Treatments with IMQ and/or allopregnanolone (3α,5α-THP) did not change TLR7 (full length and cleaved fragment) expression in hMDM from both female and male donors when compared with vehicle control (CTL). There were no differences in the levels of TLR7 (full length and cleaved fragment) between females and males at baseline or after treatments with IMQ with or without allopregnanolone (3α,5α-THP). Three-way ANOVA, Tukey's post hoc test, $p>0.05$.
